# Paediatric rotavirus vaccination, coeliac disease and type 1 diabetes in children: a population-based cohort study

**DOI:** 10.1186/s12916-021-02017-1

**Published:** 2021-06-29

**Authors:** Thomas Inns, Kate M. Fleming, Miren Iturriza-Gomara, Daniel Hungerford

**Affiliations:** 1 St Helens and Knowsley Teaching Hospitals NHS Trust, Merseyside, UK; 2 NIHR HPRU in Gastrointestinal Infections at University of Liverpool, Liverpool, UK; 3 Institute of Population Health, University of Liverpool, Liverpool, UK; 4 Department of Clinical Infection, Microbiology and Immunology, University of Liverpool, Liverpool, UK; 5 Centre for Vaccine Innovation and Access, PATH, Geneva, Switzerland; 6 Department of Clinical Infection, Microbiology and Immunology, Institute of Infection, Veterinary & Ecological Sciences, University of Liverpool, The Ronald Ross Building, 8 West Derby Street, Liverpool, L69 7BE UK

**Keywords:** Coeliac disease, Type I diabetes, Rotavirus vaccine, Vaccines, Cohort study, Survival analysis, Observation study, Infectious disease

## Abstract

**Background:**

Rotavirus infection has been proposed as a risk factor for coeliac disease (CD) and type 1 diabetes (T1D). The UK introduced infant rotavirus vaccination in 2013. We have previously shown that rotavirus vaccination can have beneficial off-target effects on syndromes, such as hospitalised seizures. We therefore investigated whether rotavirus vaccination prevents CD and T1D in the UK.

**Methods:**

A cohort study of children born between 2010 and 2015 was conducted using primary care records from the Clinical Practice Research Datalink. Children were followed up from 6 months to 7 years old, with censoring for outcome, death or leaving the practice. CD was defined as diagnosis of CD or the prescription of gluten-free goods. T1D was defined as a T1D diagnosis. The exposure was rotavirus vaccination, defined as one or more doses. Mixed-effects Cox regression was used to estimate hazard ratios (HR) and 95% confidence intervals (CIs). Models were adjusted for potential confounders and included random intercepts for general practices.

**Results:**

There were 880,629 children in the cohort (48.8% female). A total of 343,113 (39.0%) participants received rotavirus vaccine; among those born after the introduction of rotavirus vaccination, 93.4% were vaccinated. Study participants contributed 4,388,355 person-years, with median follow-up 5.66 person-years. There were 1657 CD cases, an incidence of 38.0 cases per 100,000 person-years. Compared with unvaccinated children, the adjusted HR for a CD was 1.05 (95% CI 0.86–1.28) for vaccinated children. Females had a 40% higher hazard than males. T1D was recorded for 733 participants, an incidence of 17.1 cases per 100,000 person-years. In adjusted analysis, rotavirus vaccination was not associated with risk of T1D (HR = 0.89, 95% CI 0.68–1.19).

**Conclusions:**

Rotavirus vaccination has reduced diarrhoeal disease morbidity and mortality substantial since licencing in 2006. Our finding from this large cohort study did not provide evidence that rotavirus vaccination prevents CD or T1D, nor is it associated with increased risk, delivering further evidence of rotavirus vaccine safety.

## Background

Coeliac disease (CD) is a chronic autoimmune inflammatory intestinal disease that is induced by exposure to gluten [1]. The global prevalence of CD is estimated to be 1.4%, causing a substantial burden of morbidity [1]. In England, the incidence of CD has increased over the last 30 years and is now 19.1 cases per 100,000 person-years, with incidence highest in children under 5 years of age (15.3 cases per 100,000 person-years) and older adults [2]. Type 1 diabetes (T1D) is also a chronic autoimmune disease, precipitated by destruction of insulin-producing pancreatic cells, leading to insulin deficiency [3]. Globally, an estimated 90,000 children are diagnosed with T1D each year, with incidence rates increasing over time [3]. The incidence rate of T1D for children in England and Wales was estimated to be 24.5 cases per 100,000 per year in 2018/19, with incidence peaking in those under 5 years of age and again during puberty [4, 5]. Both CD and T1D have a complex aetiology, which include strong genetic risk factors for susceptibility, as well as environmental exposures also being influential. One such possible risk factor for both is rotavirus infection.

One study in 2006 found that multiple rotavirus infections increased the risk of CD, adjusted for other risk factors [6]. This was supported by an immunological study which showed the similarity between a coeliac peptide and a key rotavirus protein [7]. Similarly, a potential biological basis for rotavirus infection increasing the risk of T1D was provided by studies in genetically-susceptible mice that showed the immune mechanism behind the acceleration of T1D by rotavirus [8]. This was further supported by an association between rotavirus seroconversion and increases in islet antibodies found in children [9], and epidemiological evidence from a cohort of children which found that an increased risk of T1D was associated with a greater number of gastrointestinal infections, such as rotavirus [10].

Considering rotavirus infection as a potential risk factor for CD and T1D, it has led to the hypothesis that rotavirus vaccination could be an effective intervention to reduce the incidence of these diseases in children. In the UK, the live attenuated rotavirus vaccine (Rotarix) was first licensed in 2006 and available on the private market, before being introduced into the paediatric immunisation programme in July 2013, with two doses delivered at 8 and 12 weeks of age [11]. Vaccine uptake is high, estimated at 90% for two doses [12]. Since introduction, there have been large reductions in laboratory detections and hospitalisations for rotavirus gastroenteritis (RVGE), as well as reductions in primary care [13, 14]. There has also been evidence that rotavirus vaccination has beneficial off-target effects, such as reducing seizures in children [15]. So far, three studies have examined the possible protective effect of rotavirus vaccination on CD; however, a recent systematic review of this topic found that these were of low quality and did not produce consistent findings [16]. Similarly, a recent narrative review found that there were differences in the small number of studies examining the association between rotavirus vaccination and T1D and that further data were required to understand this relationship [17].

In this study, we aimed to address the uncertainty in the evidence around these potential associations by researching whether children born in England between 2010 and 2015 who received rotavirus vaccination (Rotarix®) have a different risk of developing CD and T1D than those without this vaccination.

## Methods

### Study design and setting

We applied a cohort design to prospectively collected healthcare data from the Clinical Practice Research Datalink (CPRD) Aurum. CPRD Aurum contains data relating to health and healthcare utilisation from people registered at a network of general practices across the UK. This is a de-identified register of approximately 13 million current patients across the UK [18]. Patients registered in CPRD Aurum are representative of the population with regard to demographic factors, geographical distribution and level of deprivation [19]. Individual-level de-identified data were extracted from CPRD Aurum in May 2020.

### Study participants

In this study, we included children registered with CPRD Aurum who were born in the UK between 01 January 2010 and 31 December 2015 and registered at a CPRD GP practice within 14 weeks of birth. Additionally, we only included participant records which met CPRD Aurum quality standards regarding valid age, sex and registration period. In order to account for vaccine hesitancy as a potentially confounding factor, we only included children in the study with a record of DTaP/IPV vaccination [20, 21]. We used this as a proxy measure of vaccine hesitancy because this vaccine has been a long-standing part of the routine childhood immunisation schedule in the UK. All participants who met these criteria were included in the cohort.

Participants entered the follow-up period at 6 months of age (UK vaccination schedule upper limit for second dose of Rotarix®). Participants exited the cohort if they were recorded as having the outcome, or on their seventh birthday if they did not have the outcome. If the participants transferred out from the GP practice, died, were recorded as experiencing rotavirus gastroenteritis or had a last recorded data collection date before their seventh birthday, they exited the cohort.

### Study variables

In this study, the primary outcomes were CD and T1D. CD was defined based on either a recorded diagnosis of CD or the prescription of gluten-free goods [22]. T1D was defined based on a recorded diagnosis of T1D [23]. The primary exposure was rotavirus vaccination, a binary variable defined as having a record of one or more doses of Rotarix® in CPRD Aurum records. The list of CPRD Aurum codes used to classify the primary exposure variable, the two outcome variables and DTaP/IPV vaccination status for study inclusion have been published as an open-source document [24].

There were a range of potentially confounding factors for the associations between this exposure and these outcomes which we identified a priori*.* We identified healthcare-seeking behaviour as a potentially confounding factor. To adjust for this, we calculated a rate of GP consultations per person-year based on unique consultation events recorded in CPRD Aurum. We included sex as a variable, as recorded in CPRD Aurum. Geography of residence was assigned to one of ten regions of England, as used by CPRD Aurum. We adjusted for socio-economic status using English Index of Multiple Deprivation (IMD) quintiles, as assigned by CPRD Aurum using participant residence at the time of primary care registration. IMD quintiles were categorised from 1 (least deprived) to 5 (most deprived). We also used year of birth as a variable to adjust by proxy for changes in diagnosis patterns of the outcomes over time. Additionally, we used GP practice as a variable, based on the unique practice code within CPRD Aurum.

### Bias

One of the key potential biases in this study was the increased awareness and diagnostic testing for both outcomes over this time period. In order to adjust for this, we included a year of birth variable in our analysis and also conducted sensitivity analyses including only those born post vaccine introduction in 2013.

Another potential bias affecting observational studies assessing vaccines with high uptake is the bias related to healthcare access. This bias has previously been demonstrated in a study assessing rotavirus vaccine effectiveness in the UK [20]. Given that both outcomes are chronic conditions rather than an acute illnesses, they may not have been present in the same way. We assessed the potential presence of this bias by estimating the association between the rate of GP consultations and both primary outcomes (CD and T1D).

### Sample size

We estimated sample sizes in Stata 15 (StataCorp LLC: College Station, TX), using the exponential test comparing two independent hazard rates. We specified a follow-up period of 6 years, with loss to follow-up of 0.5 over 3 years, α of 0.05, using 80% power and a 1:1 ratio of comparison groups.

Prevalence of CD in children under the age of seven is estimated to be 1% [25, 26]. However, only a quarter of cases are likely to be diagnosed and a recent study estimated CD prevalence to be 0.15% [27]. For T1D in children under the age of seven in the UK, we assumed a prevalence of 0.1% [4]. Therefore, we conducted sample size calculations assuming 0.15% prevalence for CD and 0.1% for TD1. In order to observe a 20% reduction in hazard, we calculated this would require 435,470 participants for CD and 652,988 participants for T1D.

### Statistical methods

We described the characteristics of the study cohort, including the distribution of variables and person-years at-risk. We then compared the distribution of these study variables in the exposed and unexposed groups, using statistical tests of the null hypothesis that each variable was equally distributed in each group. For this, we used Chi-squared tests for categorical variables and *t*-tests for continuous variables. The explanatory variables identified a priori were sex, year of birth, rate of GP consultations per year and IMD quintile. Region of residence was also included as an explanatory variable.

The primary outcomes in this analysis were CD and T1D, both binary outcomes for each participant. We used a survival analysis to account for differential person-time at-risk and used hazard ratios (HR) as our measure of association. We used a Kaplan-Meier estimator to visualise the survival function of exposed and unexposed groups. Time at risk was defined as days since the participant entered the cohort at six months of age. We used a mixed-effects Cox regression model to estimate the relationships between the hazards of CD, T1D and a range of explanatory variables. Random GP practice-level intercepts were included to accommodate unmeasured differences in explanatory variable and outcome recording between practices. To build the multivariable model, we initially included all explanatory variables identified as scientifically relevant, then added other explanatory variables, retaining them in the model if they improved model parsimony, as measured using the Akaike Information Criterion (AIC). We tested the proportional hazards assumption of the semi-parametric Cox model and checked for interactions between explanatory variables.

In addition, we conducted a sensitivity analysis which included only those born post vaccine introduction in July 2013 in order to analyse to the effect of increased awareness and diagnostic testing in this later follow-up period. We also conducted another sensitivity analysis which did not right-censor person-time at-risk at participants’ seventh birthdays, to examine any potential impact on older children. We conducted all analyses using R version 3.6.1 (R Core Team: Vienna, Austria), using the survival and coxme packages [28, 29].

## Results

### Study participants

The study dataset extracted from CPRD Aurum contained 926,013 participants. After applying study inclusion criteria, the cohort contained 880,629 participants. Of these, 429,673 (48.8%) were female and the most common geographical locations were London (20.6%) and West Midlands (16.7%). Participants were registered at a total of 890 GP practices. A total of 343,113 (39.0%) participants had rotavirus vaccination; in those born after the introduction of routine rotavirus vaccination, 93.4% were vaccinated. Of those with rotavirus vaccination, 19,413 (5.7%) received only one dose. We also found that participants were recorded having 1317 episodes of rotavirus gastroenteritis. For a full description of cohort characteristics and explanatory variable distributions, please see Table 1.

**Table 1:** Cohort participant characteristics and distribution of explanatory variables, by rotavirus vaccination status

Variable		Overall		No rotavirus vaccination		Rotavirus vaccination		*P* value^a^
		N	***%***	n	***%***	n	***%***	
Total participants		880,629		537,516		343,113		
Sex	Male	450,956	*51.2*	275,336	*51.2*	175,620	*51.2*	0.719
	Female	429,673	*48.8*	262,180	*48.8*	167,493	*48.8*	
Year of birth	2010	162,510	*18.5*	161,049	*30.0*	1461	*0.4*	< 0.001
	2011	158,273	*18.0*	156,413	*29.1*	1860	*0.5*	
	2012	154,619	*17.6*	152,182	*28.3*	2437	*0.7*	
	2013	142,384	*16.2*	50,625	*9.4*	91,759	*26.7*	
	2014	135,007	*15.3*	9726	*1.8*	125,281	*36.5*	
	2015	127,836	*14.5*	7521	*1.4*	120,315	*35.1*	
Region	North East	34,011	*3.9*	20,654	*3.8*	13,357	*3.9*	< 0.001
	North West	130,546	*14.8*	79,505	*14.8*	51,041	*14.9*	
	Yorkshire and Humber	31,402	*3.6*	19,359	*3.6*	12,043	*3.5*	
	East Midlands	21,895	*2.5*	13,548	*2.5*	8347	*2.4*	
	West Midlands	146,977	*16.7*	89,481	*16.7*	57,496	*16.8*	
	East of England	50,765	*5.8*	30,517	*5.7*	20,248	*5.9*	
	South West	110,315	*12.5*	67,537	*12.6*	42,778	*12.5*	
	South Central	109,198	*12.4*	66,514	*12.4*	42,684	*12.4*	
	London	180,914	*20.6*	111,218	*20.7*	69,696	*20.3*	
	South East Coast	64,231	*7.3*	38,920	*7.2*	25,311	*7.4*	
IMD quintile	1	173,439	*19.7*	104,602	*19.5*	68,837	*20.1*	< 0.001
	2	158,821	*18.1*	95,364	*17.8*	63,457	*18.5*	
	3	161,999	*18.4*	98,331	*18.3*	63,668	*18.6*	
	4	182,182	*20.7*	111,907	*20.8*	70,275	*20.5*	
	5	203,415	*23.1*	126,859	*23.6*	76,556	*22.3*	
T1D record	No	879,896	*99.9*	537,027	*99.9*	342,869	*99.9*	0.001
	Yes	733	*0.1*	493	*0.1*	240	*0.1*	
CD record	No	878,972	*99.8*	536,380	*99.9*	342,592	*99.9*	< 0.001
	Yes	1657	*0.2*	1136	*0.2*	521	*0.2*	
		**Mean**	***SD***	**Mean**	***SD***	**Mean**	***SD***	
GP consultations per year		7.29	*4.96*	7.08	*5.02*	7.63	*4.84*	< 0.001
Person-days at-risk		1843	*773*	2015	*809*	1577	*624*	< 0.001

*SD* standard deviation

^a^Comparing the distribution of participants within a variable between vaccinated and unvaccinated

When we explored the relationship between rotavirus vaccination and other explanatory variables, all of these variables, apart from sex (p = 0.719), were associated with vaccine status. Due to the introduction of the rotavirus vaccine into the routine immunisation schedule in 2013, there was an association with year of birth (*p* < 0.001). Children with rotavirus vaccination also had a higher mean rate of GP consultations per year (7.63) than children without rotavirus vaccination (7.08, *p* < 0.001). There was an association between rotavirus vaccination and geographical location (*p* < 0.001) and rotavirus vaccination and IMD quintile of residence (*p* < 0.001).

Study participants contributed a total of 4,388,355 person-years at-risk, with the median follow-up period being 5.66 person-years per participant. The cohort follow-up period started on 26 March 2010 and ended on 28 April 2020. The outcome of CD was recorded for 1657 participants, an incidence rate of 38.0 cases per 100,000 person-years at-risk. The median age at diagnosis of CD was 3.2 years for participants in this cohort. T1D was recorded for 733 participants, an incidence rate of 17.1 cases per 100,000 person-years at-risk. The median age at diagnosis of T1D was 3.3 years for participants in this cohort. The two outcomes were not mutually exclusive; CD was also diagnosed in 54 of the 733 participants with T1D (7.4%)

### Survival analysis

**Fig. 1:**
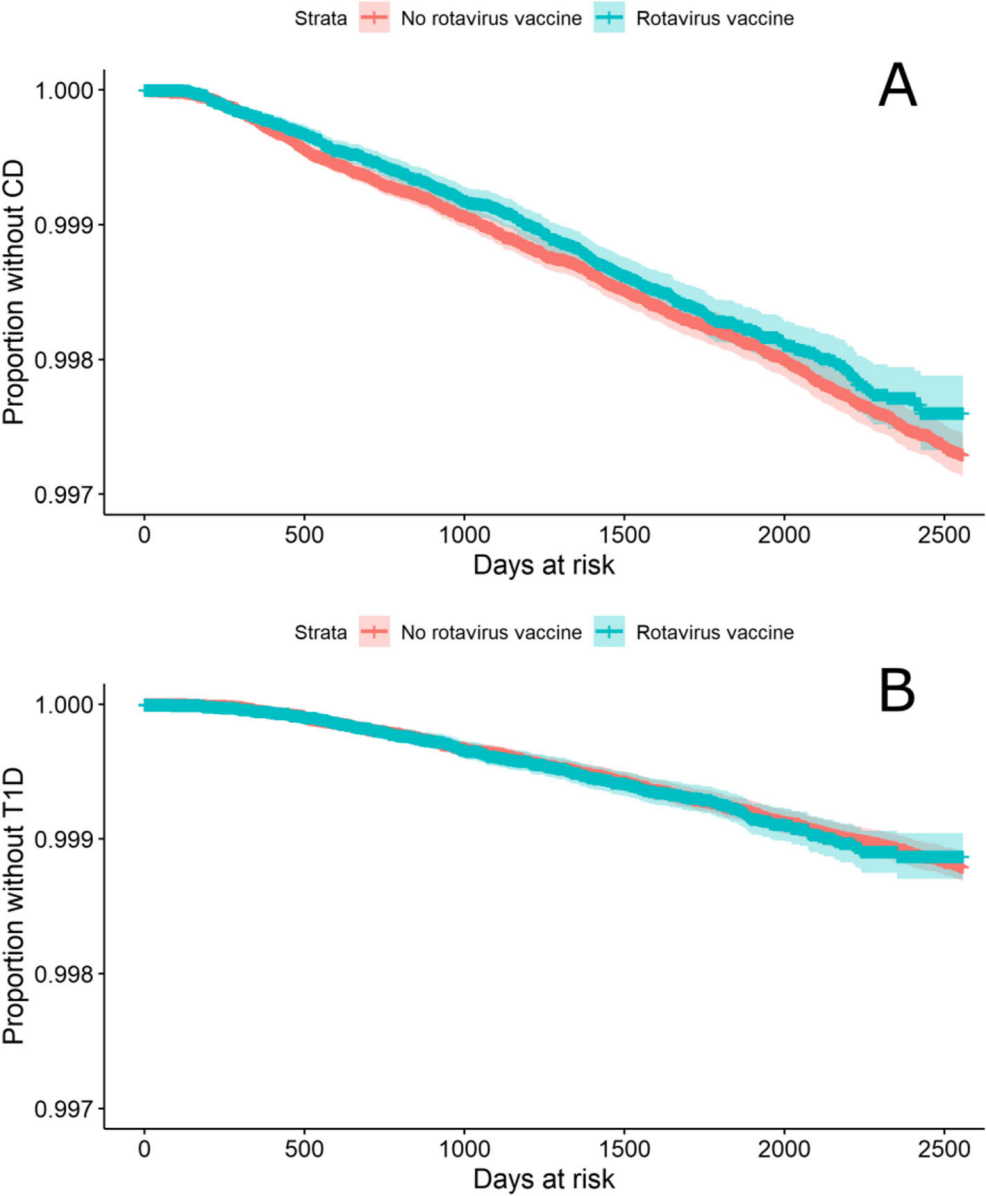
Kaplan-Meier survival estimator for **A** CD and **B** T1D, by rotavirus vaccination status

The Kaplan-Meier survival estimators for CD and T1D, stratified by rotavirus vaccination status, are shown in Fig. 1. For both CD and T1D, due to the large proportion without the outcome, calculation of median survival time was not relevant. Based on visual inspection of both Kaplan-Meier plots, the hazards in both groups were proportional.

The results of mixed-effects Cox regression modelling, with random intercepts for GP practices, are shown in Table 2. In univariable analysis the HR for the association between rotavirus vaccination and CD was 0.93 (95% CI 0.84–1.04), when simultaneously adjusted for other explanatory variables, the HR increased to 1.05 (95% CI 0.86–1.28). Sex was associated with CD in both univariable and adjusted models, with females having 40% higher hazard than males (p < 0.001). Rotavirus vaccination was not associated with T1D in either univariable analysis (HR 1.01, 95% CI 0.87–1.19), or when adjusted for confounders in multivariable analysis (HR 0.89, 95% CI 0.68–1.19).

**Table 2:** Results from mixed-effects Cox regression models, with random intercepts for GP practices

		CD				T1D			
Variable		Univariable		Adjusted		Univariable		Adjusted	
		HR	***95% CI***	HR	***95% CI***	HR	95% CI	HR	95% CI
Rotavirus vaccination	No	*ref*	*ref*	*ref*	*ref*	*ref*	*ref*	*ref*	*ref*
	Yes	0.93	0.84–1.04	1.05	0.86–1.28	1.01	0.87–1.19	0.89	0.68–1.19
Sex	Male	*ref*	*ref*	*ref*	*ref*	*Ref*	*ref*	*ref*	*ref*
	Female	1.42	1.29–1.56	1.42	1.29–1.56	1.02	0.88–1.17	1.02	0.88–1.17
Year of birth	2010	*ref*	*ref*	*ref*	*Ref*	*ref*	*ref*	*ref*	*ref*
	2011	0.98	0.85–1.14	0.98	0.85–1.14	1.08	0.86–1.34	1.08	0.86–1.34
	2012	1.03	0.89–1.19	1.03	0.89–1.19	0.93	0.74–1.17	0.93	0.74–1.17
	2013	0.91	0.77–1.06	0.88	0.72–1.08	1.11	0.88–1.41	1.19	0.89–1.58
	2014	0.95	0.80–1.13	0.91	0.71–1.17	1.15	0.90–1.48	1.28	0.89–1.82
	2015	0.84	0.70–1.02	0.81	0.6–1.02	0.89	0.65–1.19	0.98	0.66–1.46
Region	North East	*ref*	*ref*	*ref*	*ref*	*Ref*	*Ref*	*ref*	*Ref*
	North West	1.18	0.85–1.63	1.12	0.82–1.54	0.81	0.55–1.18	–	–
	Yorkshire and Humber	1.45	0.97–2.15	1.39	0.95–2.04	0.61	0.35–1.06	–	–
	East Midlands	1.07	0.68–1.70	0.96	0.61–1.50	0.91	0.52–1.57	–	–
	West Midlands	1.18	0.86–1.62	1.10	0.80–1.51	0.66	0.45–0.97	–	–
	East of England	1.23	0.85–1.78	1.01	0.70–1.45	1.08	0.70–1.66	–	–
	South West	1.25	0.90–1.74	1.13	0.82–1.56	0.83	0.56–1.23	–	–
	South Central	1.40	1.01–1.94	1.14	0.83–1.58	0.77	0.52–1.15	–	–
	London	1.06	0.77–1.46	1.02	0.74–1.40	0.63	0.43–0.92	–	–
	South East Coast	1.31	0.93–1.86	1.11	0.79–1.57	0.94	0.62–1.43	–	–
IMD quintile	1	*ref*	*ref*	*ref*	*ref*	*ref*	*Ref*	*ref*	*Ref*
	2	0.96	0.84–1.11	0.97	0.84–1.12	1.20	0.96–1.51	1.20	0.96–1.51
	3	0.86	0.75–1.00	0.87	0.75–1.02	1.05	0.83–1.33	1.05	0.83–1.33
	4	0.72	0.61–0.84	0.73	0.62–0.85	1.10	0.87–1.38	1.10	0.87–1.39
	5	0.58	0.50–0.68	0.58	0.49–0.69	1.02	0.81–1.29	1.02	0.81–1.29
GP consultations per year		1.24	1.24–1.25	–	–	1.25	1.24–1.25	–	–

Sensitivity analysis results are shown in Table 3. Restricting the cohort to only those born after 1st July 2013 had a minor effect on the point estimates. Removing censoring of follow-up time at the seventh birthday did not change the association between rotavirus vaccination and CD or the association between rotavirus vaccination and T1D.

**Table 3:** Association between rotavirus vaccination and CD disease under different sensitivity analysis conditions

	CD				T1D			
Sensitivity condition	Univariable		Adjusted		Univariable		Adjusted	
	HR	***95% CI***	HR	***95% CI***	HR	***95% CI***	HR	***95% CI***
Original analysis	0.93	0.84–1.04	1.05	0.86–1.28	1.01	0.87–1.19	0.89	0.68–1.19
Only including those born after introduction of rotavirus vaccine	1.16	0.75–1.78	1.09	0.71–1.69	0.76	0.45–1.29	0.77	0.45–1.31
Removing censoring at seventh birthday	0.92	0.83–1.03	1.03	0.84–1.26	1.01	0.86–1.19	0.90	0.68–1.18

## Discussion

In this analysis of a large general practice cohort of children followed up for a mean of 5.1 years, we found no evidence of a protective or negative effect of rotavirus vaccination on either CD or T1D.

The study findings regarding the association with CD are broadly compatible with those previous studies in other settings. Two small cohort studies previously found a protective effect of rotavirus vaccination on CD, although these studies were low-quality on the Newcastle-Ottawa scale with unclear adjustment for confounders [25, 30]. The largest cohort study, following 121,650 Finnish children over 5 years found a protective effect, but this was not significant [31]. This lack of significant association from two cohort studies in different European countries adds consistency of evidence to reject a causal association between rotavirus vaccination and risk of CD.

Regarding the study results for the association between rotavirus vaccination and T1D, our findings do not support those from an ecological study in Australia, which found that rotavirus vaccination had a protective effect on T1D [32], and another from a United States cohort study based on medical insurance data [33]. However, our results are in agreement with several recent cohort studies, two from the USA and two from Finland, which found that vaccination against rotavirus was not associated with T1D incidence in children [25, 31, 34, 35].

Although no association between rotavirus vaccination and two off-target chronic diseases, CD and T1D, were found in this study previous studies have shown evidence of the benefit of rotavirus vaccinations on other syndromes such as, childhood seizures and Kawasaki disease [15, 36, 37]. This suggests that continued investigation into off-target effects of rotavirus vaccination is of scientific and public health value.

### Study strengths

One of the key strengths of this study is that it uses CPRD Aurum data. Patients at GP practices enrolled in CPRD are representative of the population in England with regard to age, gender, geographical spread and deprivation [19]. Because this sampling frame of CRPD Aurum patients is representative of the population of England, it would be reasonable to infer that the study findings are generalisable to the wider population. The size of the CPRD Aurum database meant that it was feasible to include over 880,000 participants in this study, exceeding the calculated sample size.

Another strength of using CPRD Aurum data is the completeness and detail of information it contains. CPRD Aurum routinely undertake data validation and quality assurance work on their database [19]. This meant that we were able to exclude potential participants where their data did not meet CPRD Aurum quality standards, improving confidence in the completeness and reliability of this data source.

Regarding the use of CPRD Aurum records as a measure of CD and T1D diagnoses, a similar approach of using diagnostic codes for CD in CPRD Aurum data was used previously [2]. It is possible that this approach underestimates CD and T1D incidence by missing cases diagnosed in secondary care and entered in GP notes as free text. However as participant-level free-text records were not available from CPRD Aurum due to privacy concerns, this meant that it was not possible to formally validate this method for our diagnoses.

One trend which was relevant for this analysis is the increase in the incidence of CD diagnoses over time in England; CD increased from 5.16 cases per 100,000 in 1990 to 19.14 cases per 100,000 in 2011 [2]. Although these data do not cover most of the follow-up period in this study, from 2010 to 2020, it may be inferred that these trends have continued. Compared to CD, T1D incidence in children in England has remained stable since 2013 [4]. The increase in the rate of CD diagnoses may have affected the reliability of using CPRD Aurum as the recording as the recording of these diseases may have changed over time. This may have created a potentially confounding relationship as time was also associated with the probability of being vaccinated against rotavirus. One of the strengths of this analysis was that we attempted to adjust for this by including year of birth as an explanatory variable; however it is possible that some residual confounding remained.

Another strength of the study was the inclusion of socio-economic deprivation data. This was an important confounder as rotavirus vaccination coverage in the UK has been shown to be lower in more deprived socio-economic groups [14], and CD incidence has been found to be lower in the most deprived people [2]. This was consistent with our finding that the risk of CD was lower in those in IMD quintiles 4 and 5 (the most deprived).

### Study limitations

A key limitation of this study relates to the period of follow-up in which we measured the diagnosis of CD and T1D. In this study, we used a 7-year follow-up period; this was the maximum period for those born since the introduction of routine rotavirus vaccination in July 2013. This was relevant because the incidence of CD and T1D changes with age. CD is relatively higher in those under five, then decreases and increases again in those aged over 30 [2]. T1D incidence is low in those under 2 years of age, then increases between the ages of two and ten [38]. We used a sensitivity analysis to examine the impact of removing right-censoring at 7 years; however, as very few children born before July 2013 were exposed to rotavirus vaccine, this follow-up period of 7 years limits the inference which could be made from this study on the association between rotavirus vaccination and both CD and T1D diagnosed later in life. The impact of this 7-year follow-up period on the size and direction of results is uncertain. Should cases of CD and T1D continue to occur at similar proportions in older ages, these results would continue to apply. It could however be the case that a biological mechanism for rotavirus vaccination to protect against CD and/or T1D manifests later in life, and is not captured by this study. Subsequent follow-up of this cohort over a longer time period would be an effective method for addressing this issue.

In this study we analysed exposure as a binary variable; with exposure being based on having one or more rotavirus vaccinations. This is consistent with previous rotavirus research in England [14]; however, there are two doses recommended in the routine schedule, so it could be viewed as a limitation that we did not analyse the two “levels” of vaccination separately. Finding a dose-response relationship would have been valuable in providing evidence that a potential association is causal. However, in this study it was not possible to analyse this as the number of participants only receiving one dose was so small (5.7%).

## Conclusions

Finding a positive non-rotavirus health outcome from rotavirus vaccination would further improve vaccine uptake, reducing health inequalities in the UK, and improve the economic case for introducing routine rotavirus vaccination in other European countries, reducing the burden of rotavirus morbidity in those populations. However, in this large cohort study, we did not find evidence that in children born in England between 2010 and 2015 given rotavirus vaccination (Rotarix®) have lower rates of diagnosed CD and T1D than those without this vaccination. However, it would be valuable to repeat this analysis in the future to allow longer follow-up and understand the potential association in older children. Importantly these findings provide further evidence of the safety of rotavirus vaccination in children. This study advocates for further observational studies investigating off-target effects of paediatric rotavirus vaccines in diverse settings.

## Data Availability

Data from this study is not available from the authors. Study data is available through formal request from CPRD.

## Abbreviations

AIC: Akaike Information Criterion
CPRD: Clinical Practice Research Datalink
CD: Coeliac disease
HR: Hazard ratio
NHS: National Health Service
*SD*: Standard deviation
T1D: Type 1 diabetes
UK: United Kingdom
